# Evaluating fatty acid profiles in anisakid nematode parasites and adjacent tissue of European hake (*Merluccius merluccius*): a first insight into local host-parasite lipid dynamics

**DOI:** 10.1007/s00436-025-08477-1

**Published:** 2025-03-13

**Authors:** João P. Monteiro, Tiago Sousa, Marisa Pinho, Fernando Atroch, Luís Filipe Rangel, Camilo Ayra Pardo, Maria João Santos, Rita Barracosa, Felisa Rey, M. Rosário Domingues, Ricardo Calado

**Affiliations:** 1 CESAM & Departamento de Química, Universidade de Aveiro, Campus Universitário de Santiago, 3810-193 Aveiro, Portugal; 2 Centro de Espetrometria de Massa & LAQV-REQUIMTE & Departamento de Química, Universidade de Aveiro, Campus Universitário de Santiago, 3810-193 Aveiro, Portugal; 3 CIVG – Vasco da Gama Research Center / EUVG, Vasco da Gama University School, Coimbra, Portugal; 4 Division of Aquaculture, Upgrading and Bioprospection, Portuguese Institute for the Sea and CIIMAR, Departmento de Biologia, Faculdade de Ciências, Universidade do Porto, Rua do Campo Alegre s/n, FC4, 4169-007 Porto, Portugal; 5 CIIMAR, Universidade do Porto, Terminal de Cruzeiros do Porto de Leixões, Av. General Norton de Matos, s/n, 4450-208 Matosinhos, Portugal; 6 MC Shared Services S.A. - Rua João Mendonça, 529, 4464-501 Senhora da Hora, Matosinhos, Portugal; 7 ECOMARE & CESAM & Departamento de Biologia, Universidade de Aveiro, Campus Universitário de Santiago, 3810-193 Aveiro, Portugal

**Keywords:** Anisakid nematode parasites, Fatty acids, Fish parasitism, Food safety, Lipids, Nutritional quality

## Abstract

Parasitism in fish is a widespread and ecologically significant phenomenon, affecting fish in both wild and aquaculture environments. Comprehending parasitism is essential for managing fish populations, protecting fish health, and preventing human exposure to zoonotic parasites. Understanding lipid dynamics between parasitic organisms and their hosts is crucial for elucidating host-parasite interactions. Although the third larval (L3) stage of anisakid larvae is not a developing stage, and therefore not as dependent on the host for the acquisition of nutrients, there are hints of interplay between parasites and fish hosts, also in terms of lipid content. This study aimed to characterize for the first time the fatty acid profiles of anisakid nematode parasites and adjacent tissue in the European hake (*Merluccius merluccius*) in order to shed some light on these intricate relationships. Fatty acid analysis revealed significant differences in the percentages of individual fatty acids between anisakid nematodes and adjacent European hake tissue. Anisakids presented a higher content in stearic (18:0), vaccenic (18:1*n*-7), and linoleic (18:2*n*-6) acids, while in turn, the belly flap tissue of the fish presented significantly higher contents in palmitic (16:0) and especially docosahexaenoic acid (22:6*n*-3) than the parasite. These differences suggest unique lipid metabolic pathways between parasite and fish, and that parasitism and the possible acquisition of lipids from the host (hake) do not profoundly shape the fatty acid profile of the parasites. Furthermore, the distinct fatty acid signatures described for parasites and hosts may serve as baselines to follow possible changes in the ecological statuses of both species and even to appraise the nutritional features of European hake when affected by parasitism. This study provides valuable insights into the lipid dynamics within host-parasite systems and underscores the importance of further research to unravel the complexities of these interactions.

**Supplementary Information:**

The online version contains supplementary material available at 10.1007/s00436-025-08477-1.

## Introduction

Parasitism is a biological relationship in which one organism, the parasite, benefits at the expense of a host. The impact on the host varies depending on the type of parasite and the severity of the infestation, which can include reduced nutrient absorption, tissue damage, weakened immune functions, or behavioral changes (Frank and Schmid-Hempel 2008; Sastry and Bhat 2018). The diverse range of parasitic organisms, each with unique life cycles and effects on their hosts, highlights the complexity of parasitic interactions within aquatic ecosystems (Marcogliese 2008, 2016; Lymbery et al. 2020). The rise in parasitism in fish can be attributed to various factors, including changes in environmental conditions, aquaculture practices, and global trade (Marcogliese 2008; Lõhmus and Björklund 2015; Cable et al. 2017; Cascarano et al. 2021; Williams et al. 2020, 2022, 2023; Buchmann 2022; Madsen and Stauffer 2024). These factors have contributed to the proliferation of anisakid nematodes in marine ecosystems (Fiorenza et al. 2020), leading to higher incidences of parasitic infections in commercially important fish species, and in the European hake in particular (Pascual et al. 2018; Santos et al. 2022; EFSA Panel on Biological Hazards 2024). Consequently, the fishing industry faces challenges in ensuring the quality and safety of fish products, as well as potential economic losses due to rejected batches of fish due to parasitic contamination (Shinn et al. 2015; Bao et al. 2018, 2019; Caldeira et al. 2021).

Anisakid nematodes belong to phylum Nematoda, order Rhabditida, and family Anisakidae (according to WORM’S https://marinespecies.org/aphia.php?p=taxdetails&id=19961). The Anisakidae comprises the most important and relevant zoonotic species, belonging to the genera *Anisakis*, *Phocanema*, and *Contracaecum* (EFSA Panel on Biological Hazards 2024). Anisakid nematodes are non-host-specific parasites able to infect numerous marine animal species, including several economically important fish (Aibinu et al. 2019; Debenedetti et al. 2019; Ozuni et al. 2021). Hake is among the fish species that may act as paratenic hosts for anisakids, including the European hake (*Merluccius merluccius*) in particular, reported as one of the most infected fish species worldwide (Valero et al. 2006; Farjallah et al. 2008; Vidacek et al. 2009; Ferrer-Maza et al. 2014; Cipriani et al. 2015; Cipriani et al. 2018; Abou-Rahma et al. 2016; Aibinu et al. 2019; Fuentes et al. 2022; Santos et al. 2022; Jouini et al. 2023; Ramilo et al. 2023). The presence of anisakid parasites can compromise the quality and safety of fish products destined for human consumption (Buchmann and Mehrdana 2016; Horbowy et al. 2016; Hussein et al. 2022). With the increasing popularity of raw and undercooked seafood consumption worldwide, the risk of anisakid infections in humans has become a pressing public health issue (Adams et al. 1997; Bao et al. 2017; Fuentes et al. 2022; Golden et al. 2022, 2023).

Fish represent paratenic hosts for these parasites, acquiring anisakids in their third larval (L3) stage through predation and ingestion of small intermediate host crustaceans or teleostean transport hosts (Buchmann and Mehrdana 2016; Murrell 2017). In fish hosts, larvae may become encapsulated and encyst in host tissue, namely on the intestines and other visceral organs, and they do not develop further (than the third stage of larval development in which they are acquired) awaiting transmission to their definitive hosts, such as marine mammals (e.g., dolphins, seals, and whales), through the food chain (Murrell 2017; Aibinu et al. 2019). Upon fish *post-mortem* conditions, L3 larvae are known to migrate to the fish tissue (Cipriani et al. 2024; Kumas et al. 2024), including that of European hake (Audicana and Kennedy 2008; EFSA Panel on Biological Hazards 2024), representing a possible threat to the quality of the fish flesh. Moreover, during starvation periods, L3 larvae may also migrate to the muscle of the fish, particularly to belly flaps (Smith and Hemmingsen 2003; Berland 2006; Levsen et al. 2022). Nevertheless, the impact of the encysting process on fish muscle quality and the possible role of lipids in this process remain to be addressed.

Host-parasite interactions are intricate ecological relationships shaped by a multitude of factors, including inter-organism nutritional dynamics (Khan 2012; Lange et al. 2014; Frantz et al. 2018). Among these, the lipid composition of both parasites and their hosts plays a significant role in influencing parasite development and host fitness, along with host immune responses (Aitzetmüller et al. 1994; Bize et al. 2008; Arts and Kohler 2009; Vallochi et al. 2018). Moreover, the lipid interplay between parasites and hosts may occur in a way that assures the maintenance of proper membrane fluidity and permeability and the establishment of a functional barrier guaranteeing the functionality and viability of the parasites (Aitzetmüller et al. 1994; Mika et al. 2010). Although *Anisakis* spp. at the L3 stage are not in a developing stage, and therefore not as dependent on fish paratenic hosts for nutrient acquisition, reports suggest that lipid exchange occurs between parasites and fish hosts at the peritoneum level (Mika et al. 2010). Additionally, the presence of L3-stage anisakids may lead to a decline in the fish’s condition, ultimately affecting its nutritional quality (Buchmann and Mehrdana 2016). The extent of these interactions between anisakids and fish hosts and their impact in terms of nutritional quality remain mostly unstudied.

Considerable research has focused on the epidemiology, pathology, and molecular biology of anisakid infections; however, relatively little attention has been paid to the lipid dynamics involved in local host-parasite relationships. Understanding the differences between the fatty acid profiles of anisakid nematodes and their host tissues could be crucial for revealing the local interplay in terms of lipid exchange and the impact of the encysting process on the hosts’ lipid composition.

The present study aims to characterize, for the first time, the fatty acid profiles of anisakid nematode parasites and adjacent tissue in the visceral cavity of European hake, where the parasites preferentially accumulate and encyst. By elucidating the differences in fatty acid composition between parasites and host tissue, it will be possible to gain insights into the nutritional dynamics, metabolic adaptations, and ecological interactions underlying these complex host-parasite relationships.

## Materials and methods

### Chemicals

All solvents used were of HPLC grade. Chemicals were purchased from Sigma-Aldrich (Steinheim, Germany) and had a purity higher than 95%. Milli-Q water (Synergy®, Millipore Corporation, Billerica, MA, USA) was used. The 37 Component FAME Mix from Supelco (Sigma-Aldrich, St. Louis, MO, USA) and the internal standard methyl nonadecanoate (≥ 99% purity) were purchased from Sigma-Aldrich (St. Louis, MO, USA).

### Samples

**Fig. 1:**
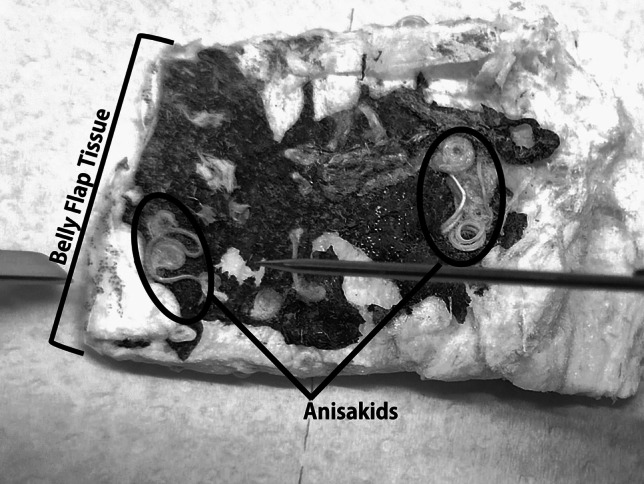
Collection of anisakids from the surface of the European hake (*Merluccius merluccius*) belly flap tissue

Five European hake (*M. merluccius*) specimens from the Bay of Biscay region were analyzed for the presence of anisakids. Fish were provided after evisceration, which impaired the sexing of the specimens that were surveyed. The specimens used in the study belonged to a rather uniform group in terms of size, since they had been previously subjected to a sorting and categorization process common to all hake specimens destined for the retail market (as they were supplied by a retail company). Moreover, we selected specimens with comparatively high parasitism levels, so as to have enough anisakid specimens attached to the tissue to perform our determinations (Table 1). A belly flap from each sampled fish was analyzed to determine the density of anisakids. Each belly flap was first cleaned from integument and spines and subsequently weighed. The number of anisakids in these clean portions of the belly flap muscle was recorded and divided by the weight of the clean belly flap muscle, in order to obtain the number of these parasites per gram of clean belly flap muscle. It is important to mention that it was not possible to obtain non-parasitized fish given the high occurrence of parasitism observed, as all fish made available to perform the present study displayed anisakids in their tissues. Samples from the belly flap, which is the tissue adjacent to the visceral cavity of the fish where anisakids preferentially accumulate, were dissected from European hake specimens and stored at − 20 °C until freeze-drying. Parasites located at the surface of each tissue sample (or slightly encysted at the surface) were collected and weighed (Fig. 1), along with the respective adjacent fish tissue.

**Table 1:** Description of the European hake specimens used in the study

Hake specimen	Size (length, mm)	Number of anisakids per gram of belly flap tissue
1	670	6.44
2	680	7.33
3	685	7.10
4	650	7.32
5	655	6.14

### Lipid extraction

Total lipid extracts of both parasites and European hake tissue were obtained by using the Bligh and Dyer method (Bligh and Dyer 1959) with slight adaptations (Bao et al. 2017). Samples (circa 10 mg of freeze-dried parasite or fish tissue) were thoroughly minced and homogenized with a mortar and pestle. Tissue was re-suspended in 1 mL of water, with the homogenized sample being transferred to glass tubes and kept on ice. Then, 2.5 mL of methanol and 1.25 mL of dichloromethane were added, followed by vigorous homogenization (vortexing for 2 min) and by a step of gentle shaking for 30 min on ice. Another 1.25 mL of dichloromethane and 2.25 mL of Milli Q water were then added, promoting a separation into two phases. The samples were then centrifuged at 626 × g for 10 min at room temperature. The lower organic phase was then collected into a new glass tube, while the aqueous phase was re-extracted by adding 1.88 mL of dichloromethane, followed by a new centrifugation at 626 × g for 10 min. The resulting organic phase was added to the one collected before and then dried under a nitrogen stream and preserved at − 20 °C for further analysis. Total lipid content in parasite or tissue samples was estimated by gravimetric analysis.

### Phospholipid quantification in total lipid extracts

The quantification of phospholipids in total lipid extracts was performed by a version of the Bartlett and Lewis method (Bartlett and Lewis 1970) with slight adaptations (Monteiro et al. 2021). Samples were dissolved in 300 µL dichloromethane, and small aliquots (10 µL, in duplicates) were transferred to acid-washed glass tubes and dried under a nitrogen stream. A volume of 125 µL of perchloric acid (70%, m/V) was added to each sample containing tube, and these were incubated for 60 min at 180 °C in a steel heating block. After hydrolysis, 850 µL of water, 125 µL of ammonium molybdate (2.5%, m/V), and 125 µL of ascorbic acid (10%, m/V) were added to each tube, and the resulting mixture was vortexed and incubated for 10 min at 100 °C in a water bath. A calibration curve was obtained by preparing standards with phosphorous concentrations ranging from 0.1 to 2.0 µg of (from a standard solution of NaH_2_PO_4_·2H_2_O, containing 100 µg of phosphorus per mL). Absorbance was measured at 797 nm, in a microplate UV–vis spectrophotometer Thermo Scientific Multiskan Go.

### Gas chromatography–mass spectrometry (GC–MS)

The fatty acid content in lipid extracts was analyzed by GC–MS after transmethylation. Aliquots of 30 µg of the total lipid extracts were transferred to glass tubes and dried under a nitrogen stream. The lipid films were dissolved in 1 mL of *n*-hexane containing the fatty acid C19:0 as internal standard (1 µg mL^−1^, CAS number 1731–94-8, Merck, Darmstadt, Germany). A volume of 200 µL of a solution of potassium hydroxide (2 M) in methanol was added to each tube, and the mixture was vortexed for 2 min to obtain the fatty acid methyl esters (FAMEs). Afterwards, 2 mL of a saturated solution of sodium chloride was added, and the resulting mixture was centrifuged for 5 min at 626 × g, promoting phase separation. Cholesterol in the upper (organic phase) was removed using a protocol available at the Lipid Web (https://lipidhome.co.uk/ms/basics/msmeprep/index.htm). A 1-cm silica column in a pipette tip with wool was pre-conditioned with 5 mL of *n*-hexane, and methyl esters were added to the top of the column and recovered by elution with *n*-hexane to diethyl ether (95:5 V/V, 3 mL), and completely dried under a nitrogen current. Finally, FAMEs, without cholesterol, were dissolved in 100 µL *n*-hexane, and 2 µL of the resulting solution was injected into an Agilent Technologies 8860 GC System (Santa Clara, CA, USA) equipped with a DB-FFAP column with 30 m length, an internal diameter of 0.32 mm, and a film thickness of 0.25 µm (J&W Scientific, Folsom, CA, USA). The GC was connected to an Agilent 5977B Network Mass Selective Detector, set to operate with an electron impact ionization at 70 eV and scanning the mass range of *m/z* 50–550 in a 1-s cycle, in a full scan mode acquisition. The oven temperature was programmed at 58 °C for 2 min, 25 °C min^−1^ to 160 °C, 2 °C min^−1^ to 210 °C, and 30 °C min^−1^ to 225 °C (held for 10 min). The initial oven temperature was set for 58 °C maintained for 2 min, followed by three successive linear increments to 160 °C at 25 °C min^−1^, to 210 °C at 2 °C min^−1^, and to 250 °C at 30 °C min^−1^. The final temperature was set at 250 °C for 10 min. The injector and detector temperatures were 220 and 280 °C, respectively. The carrier gas was helium, flowing at a rate of 1.4 mL min^−1^. Fatty acid identification was performed by comparing the retention times to those of the commercial FAME standards in the Supelco 37 Component FAME Mix (ref. 47,885-U, Sigma-Aldrich, Darmstadt, Germany) and by MS spectrum comparison with chemical databases (Wiley 275 library and AOCS lipid library). The relative percentages of fatty acids were calculated by the percent relative area method with proper normalization using internal standard methyl nonadecanoate (C19:0) and considering the sum of all relative areas of identified fatty acids. Indexes were also calculated following fatty acid determinations, namely average chain length (ACL), double bond index (DBI), peroxidizability index (PI), content in monounsaturated fatty acids (MUFA), polyunsaturated fatty acids (PUFA), polyunsaturated fatty acids *n*–3 (PUFA *n*–3), and polyunsaturated fatty acids *n*–6 (PUFA *n*–6), as previously described (Monteiro et al. 2021).

### Statistical analysis

Statistical analysis was performed using GraphPad Prism version 7.00 for Windows (GraphPad Software, La Jolla, CA, USA) by performing a Shapiro–Wilk normality test, and the existence of statistically significant differences was assessed using the Mann–Whitney *U*-test. Differences with *p* value < 0.05 were considered statistically significant. All experimental data are shown as mean ± standard deviation (SD) for five samples from each group (*N* = 5).

Principal component analysis (PCA) was performed to visualize the general 2D clustering of the same species in terms of their differences in the fatty acid profile. The data matrix was log transformed, and a new matrix was assembled using the Euclidean distance. Differences in fatty acid profiles between anisakids and adjacent hake belly flap tissue were explored using the dissimilarity percentages routine (SIMPER), and the fatty acids contributing > 60% of the difference between groups were plotted in the PCA graph.

## Results

### Lipid and phospholipid contents

**Fig. 2:**
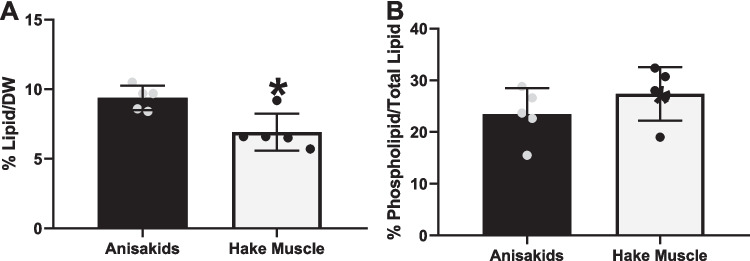
Total lipid percentage (**A**) and percentage of phospholipids (from total lipid) (**B**) in anisakids and European hake (*Merluccius merluccius*) muscle samples (*p ≤ 0.05)

Gravimetrical determination of the total lipid content in anisakids and European hake belly flap samples after lipid extraction showed that the parasites displayed a higher lipid content than fish muscle (Fig. 2A). In anisakids, lipid content amounted to 9.4 ± 0.4% of dry weight (DW), while the lipid percentage in the respective belly flaps accounted for only 6.9 ± 0.6% of DW. However, the percentage of phospholipids in total lipid content was not significantly different between parasites and adjacent hake muscle, representing about a quarter of the total lipid content in both cases (Fig. 2B).

### Fatty acid profiles

The fatty acid profiles of anisakids and the adjacent muscle were characterized, and a total of 29 fatty acids were identified and quantified in the samples (28 different fatty acids in the anisakids and 28 in the European hake muscle). Most fatty acids were present in both the parasite and the host fish, with the exceptions of behenic acid, which was only present in anisakid samples, and a 22:1 fatty acid (of which we were unable to discern the position of the unsaturation) in the fish tissue.

**Fig. 3:**
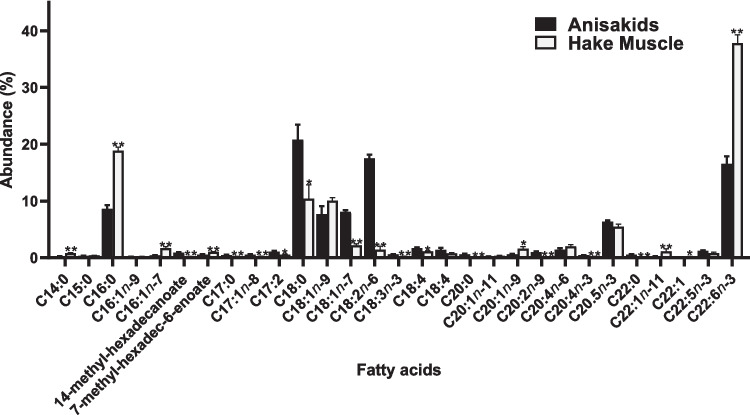
Fatty acid profiles of anisakids and the adjacent belly flap tissue in European hake (*Merluccius merluccius*) (*p ≤ 0.05; **p ≤ 0.01)

**Fig. 4:**
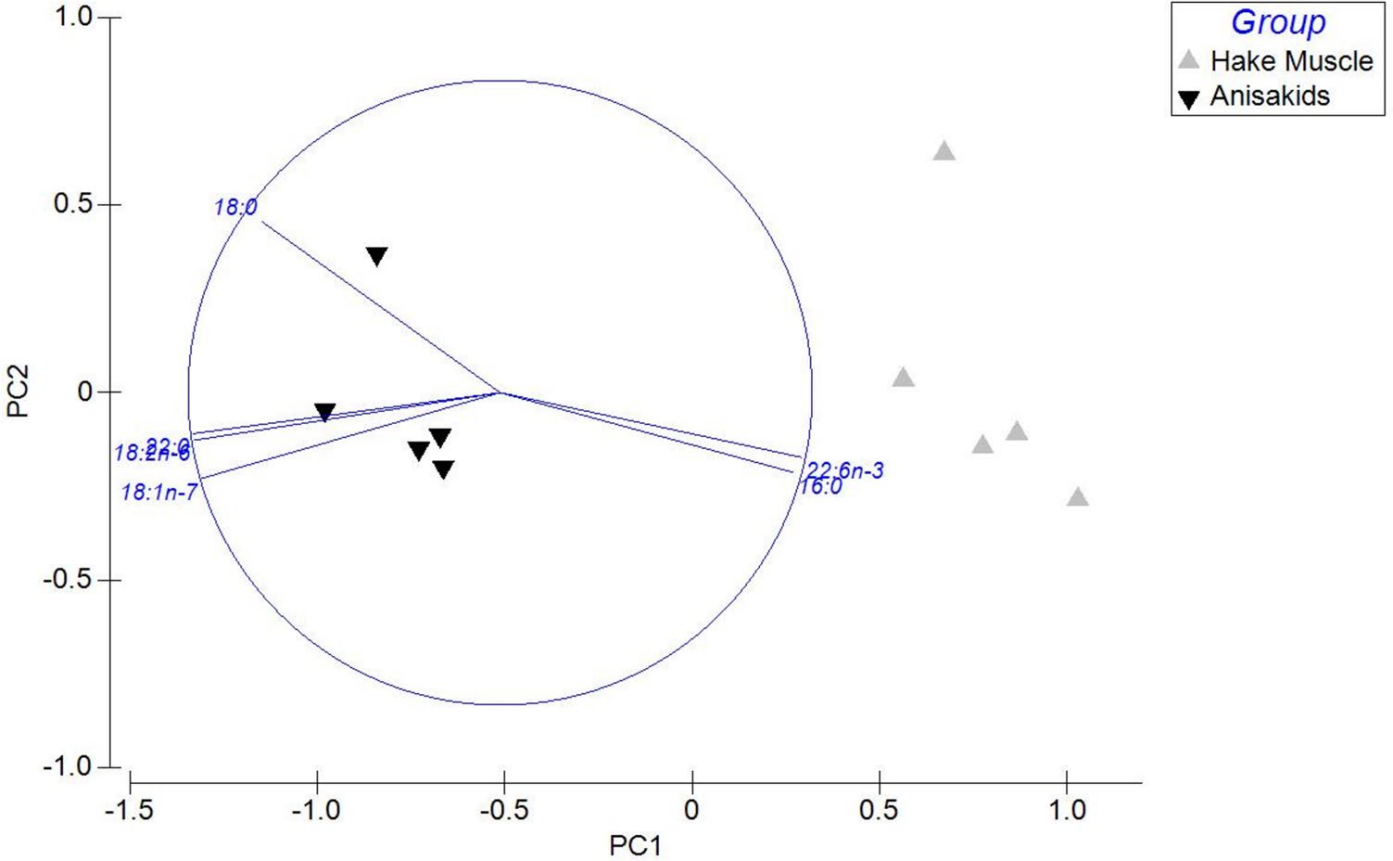
Principal component analysis (PCA) of the fatty acid profiles of anisakids and the adjacent belly flap muscle in European hake (*Merluccius merluccius*). The fatty acids contributing > 60% of the difference between groups as identified after SIMPER analysis were plotted in the PCA graph

The fatty acid composition of parasites and host tissue presented very obviously distinct compositions (Fig. 3). The most obvious differences were significantly higher contents in stearic (18:0), vaccenic (18:1*n*−7), and linoleic (18:2*n*−6) acids in the parasites than in fish tissue, while fish adjacent muscle presented higher contents in palmitic (16:0) and especially docosahexaenoic acid (DHA, 22:6*n*−3) than in parasites. Stearic (20.8 ± 2.7%) and linoleic (17.5 ± 0.7%) acids were, in fact, the most abundant fatty acids in anisakids, while in the belly flaps of European hake specimens, DHA was by far the most abundant fatty acid (37.8 ± 1.5%), followed by palmitic acid (18.9 ± 0.6%). The fatty acid profiles characterized for anisakids and adjacent belly flap muscle allowed a very clear statistical discrimination between groups by performing a principal component analysis (PCA), describing 92.2% of the total variance, including principal component 1 (82.5%) and principal component 2 (9.7%; Fig. 4). This analysis confirms the fatty acids that contribute the most to the separation between anisakid and hake belly flap samples after SIMPER analysis (Fig. 4; Supplementary Table S1).

Physiologically relevant indexes derived from fatty acid composition were determined, such as the total percentage of SFA, unsaturated (UFA), MUFA and PUFA, the UI and PI indexes, the saturated/unsaturated fatty acids (SFA/UFA) and the *n*−6/*n*−3 ratios, and the ACL (Table 2). The differences between anisakids and adjacent European hake tissue were also obvious when taking these parameters into account. European hake tissue was much richer in *n*−3 fatty acids (especially in DHA) than parasites, although the overall composition in PUFAs was similar between samples of both groups. The SFA/UFA ratio and the UI index were much higher in European hake belly flaps than in anisakids, although PUFA content was comparable between samples, meaning that PUFAs in European hake tendentially present a higher unsaturation number, which should be related to the remarkably high content in DHA in the fish. Finally, European hake belly flap tissue also tendentially presented fatty acids with longer carbon chains, as observed by differences in ACL values. Curiously, no statistical differences were reported for the contents in SFA, MUFA, and PUFA between parasites and adjacent European hake tissue.

**Table 2:** Indexes/factors derived from the fatty acid profiles of anisakids and adjacent European hake (*Merluccius merluccius*) belly flap tissue

Index/factor	Anisakids	European hake muscle
*n*−3	25.5 ± 1.6%	45.0 ± 1.5%**
*n*−6	19.2 ± 0.7%	3.5 ± 0.9%**
*n*−6/*n*−3	0.77 ± 0.06	0.08 ± 0.02**
SFA	32.1 ± 2.2%	31.2 ± 2.2%
MUFA	18.1 ± 1.4%	17.7 ± 1.4%
PUFA	49.9 ± 1.7%	51.1 ± 1.5%
SFA/UFA	0.48 ± 0.05	0.46 ± 0.05
UI	218.6 ± 9.2	300.4 ± 8.4**
PI	221.8 ± 11.6	363.6 ± 11.2**
ACL	18.8 ± 0.1	19.3 ± 0.1**

*ACL*, average chain length; *MUFA*, monounsaturated fatty acids; *PI*. peroxidizability index; *PUFA*. polyunsaturated fatty acids; *SFA*. saturated fatty acids; *UI*. unsaturation index; *UFA*. unsaturated fatty acids (**p ≤ 0.01)

## Discussion

In humans, larval anisakid nematodes (specifically *Anisakis simplex* and *Anisakis pegreffii*) (Mattiucci and Nascetti 2008; Mattiucci et al. 2013; Mattiucci et al. 2018) may cause zoonotic disease by consumption of raw or undercooked fish, a condition referred to as “anisakiasis,” caused exclusively by nematodes of genus *Anisakis* or “anisakidosis” (Mattiucci and Nascetti 2008; EFSA Panel on Biological Hazards 2024). Cases of anisakidosis are increasing worldwide (Bao et al. 2017; Aibinu et al. 2019), and clinical symptoms may vary from irritation of the esophagus and stomach, to nausea, vomiting, and diarrhea and even severe epigastric and abdominal pains (Buchmann and Mehrdana 2016; Ganucci Cancellieri et al. 2023). However, the most concerning outcome of anisakid infections in humans is reported to be allergic sensitization, ranging from urticaria to anaphylactic shock (Villazanakretzer et al. 2016).

Studies in *Anisakis* spp. have suggested that its L3 stage, the stage primarily found in paratenic fish hosts, could represent essentially a non-developing and apparent non-feeding stage (Mladineo et al. 2019). It has been hypothesized that anisakids could utilize embryonically stored nutrients (although in this case, they should decrease in size), enter a state of hypobiosis, or absorb nutrients through the cuticle while within the paratenic host, as described for other larval nematodes (Munn and Munn 2002; Mladineo et al. 2019). However, an interplay and acquisition of lipids from fish hosts at this stage has also been suggested (Mika et al. 2010). Moreover, it has also been shown for *A. simplex* that its L3 stage distribution is governed by the availability of nutrients in fish and lipids in particular (Strømnes and Andersen 1998). Parasites at this stage display a tendency to choose regions with high lipid concentrations, locally resulting in a positive correlation between larval abundance and lipid content (Strømnes 2014). This should be proof that host lipid sources should be, in fact, physiologically important for anisakid parasites.

The presence of L3 anisakids leads to a decline in the overall condition and quality of fish hosts (Buchmann and Mehrdana 2016), causing severe health effects such as tissue deformation and hemorrhages (Levsen and Berland 2012). This includes the “red vent syndrome” which is characterized by hemorrhages and inflammation around the vent (Beck et al. 2008; Noguera et al. 2009). These effects highlight the need for further investigation in order to elucidate parasite-host interactions at this stage. Moreover, the ability of anisakid larvae to penetrate the stomach wall and establish themselves in the peritoneal cavity, visceral organs, and musculature of fish hosts (Levsen and Lunestad 2010; Mehrdana et al. 2014) underscores the need for a thorough screening of the mechanisms involved in this encapsulation. Anisakids release esterases/lipases (Bahlool et al. 2013), but also proteases (Audicana and Kennedy 2008), contributing to the degradation of the surrounding tissues and facilitating larval invasion, which should influence local (lipid) composition and quality. Both the threats to human health and the potential impact on fish quality can undermine consumer confidence and negatively affect the safety, quality, and marketability of fish products.

In anisakids, a quarter of their total fatty acid content (25.5 ± 1.6%) resulted from *n*−3 fatty acids. This may be physiologically relevant, especially taking into account that eicosapentaenoic acid (EPA, 20:5*n*−3), docosapentaenoic acid (22:5*n*−3), and DHA (22:6*n*−3) have been proposed to be important for the reproductive success, development, and somatic growth of other different marine parasites (Arendt et al. 2005). The absence of *n*−3 fatty acids, particularly those of marine origin (EPA and DHA) in other helminths parasitizing land-based animals (Smith et al. 1996; Mondal et al. 2009; Ghosh et al. 2010; Becker et al. 2017), suggests that the parasitic relationship and the nutrient flux from host to parasite should significantly the parasite’s fatty acid profile. Further studies, characterizing the fatty acid composition of eggs and earlier larval stages of anisakids, will be very informative to evaluate the extent of the dependence of the fatty acid profile of parasites on the compositional features of hosts as opposite to maternal investment in the transfer of these specific fatty acids. Comparing the fatty acid profiles of anisakids with the ones of crustacean hosts (maturation from the L2 to L3 stages) should also be informative about the extent of the dependence of the fatty acid profile of anisakids on the composition of its host. Nevertheless, the evident differences observed towards the fatty acid profile of host fish surveyed in the present study (the European hake) adjacent tissue should provide proof of the existence of internal homeostatic regulatory mechanisms capable of maintaining a stable composition favorable to parasites, or of a limited dependence of the acquisition of lipids from the host at this larval stage. Some studies have suggested that the fatty acid profile of other marine fish parasites (such as copepods and acanthocephalans) largely reflects that of their host fish (Aitzetmüller et al. 1994; Tocher et al. 2010; Telahigue et al. 2017; Hajji et al. 2021). On the other hand, other reports indicated that parasites (such as acanthocephalans) have their own particular fatty acid fingerprints (Taraschewski et al. 1995), with the latter case being supported by our findings in the case of L3 stage anisakid when parasitizing fish.

Some important differences were detected between the fatty acid content of parasites (anisakids) and their host (the European hake), including major differences in some saturated and monounsaturated fatty acids. Interestingly, stearic acid and vaccenic acid, two fatty acids that are much more abundant in anisakids than in European hake belly flaps, were reported to be involved in the breaking through the host cell membranes and facilitating the successful penetration and migration inside the hosts (Ward 1982; Polak et al. 2023). In fact, the presence of stearic acid and oleic acid (although this one is not enriched in the parasite with regard to the adjacent fish tissue) was suggested to be particularly important for the invasion and establishment of infection in helminth species (Yeshi et al. 2020, 2022). One of the most interesting differences between the fatty acid composition of anisakids and adjacent belly flaps of European hake would be the fact that behenic acid (22:0), despite being a minor fatty acid, was only detected in parasites. However, it must be highlighted that other studies have reported this fatty acid in *M. merluccius* muscle (Soriguer et al. 1997; Jouini et al. 2023).

The differences observed in indexes derived from fatty acid composition may also have a physiological meaning. A lower average chain length of fatty acids should imply greater membrane fluidity and permeability (Huang et al. 1974), which may be important for the infection process and for nutrient acquisition from the host. In turn, a lower peroxidability index could be strategical to render parasites less vulnerable to lipid peroxidation (Pamplona et al. 1998) and thus to escape damage due to oxidative stress or even to increase their overall resilience.

The fact that the fatty acid profiles between parasite and host muscle tissue are so obviously dissimilar opens the perspective of using fatty acid profiling for detecting anisakid parasitism in fish, especially in cases analyzing *post-mortem* tissue samples with encysted parasites. In fact, besides encysting in starvation conditions, the *post-mortem* migration of *Anisakis* spp. from fish viscera to the muscle has also been reported in many standard conservation conditions (Bao et al. 2017). In this case, the fatty acid muscle tissue containing parasites should shift towards the characteristic features of parasite composition. In fact, a study suggested that parasite intensity may influence the degree of impact on the nutritional composition of fish, therefore reinforcing the feasibility of using fatty acid profiles to detect parasitism in fish tissues (Jouini et al. 2023). The development and improvement of the available methods for the detection and quantification of anisakid nematodes in fish and fishery products is one of the objectives of the establishment of national reference laboratories more specifically directed to the study of parasites threats, as premised EU regulation 2017/625 (European Parliament 2017; Klapper et al. 2021). Overall, lipid profiling may contribute to such an endeavor and advance the state of the art on this topic.

This is the first time that the fatty acid profile of anisakids is characterized, aside from another study using a very different technical approach and specifically focusing on the cuticular lipids of *A. simplex* and comparing those to the tissue (peritoneum) of its host, the Atlantic cod *Gadus morhua* (Mika et al. 2010). The fatty acid profile identified in this study differs from that reported in our present work, particularly with the presence of free highly unsaturated odd-number fatty acids, or the occurrence of triglycerides containing very short acyl chains (Mika et al. 2010). Although differences between parasite and host tissue were reported in that study, comparing those results with ours is challenging due to the unique characteristics of the cuticle tissue, which creates a notable disparity in the fatty acid content regarding the whole parasite profile. There is an available study comparing the proximate composition and fatty acid profiles of fillets of unparasitized and parasitized *M. merluccius* specimens (Jouini et al. 2023). This study reported a similar fatty acid profile to what we report for belly flaps, with docosahexaenoic acid clearly being the most abundant fatty acid present, followed by palmitic acid; nonetheless, in our case, we generally report a higher content in PUFAs. This work also states that proximate composition and fatty acid profiles were only affected in female specimens (which were generally more parasitized than males in that study) (Jouini et al. 2023). In this case, arachidonic acid (20:4*n*−6) and eicosapentaenoic acid (20:5*n*−3) were found to be present in lower levels in the fillets of parasitized female European hake specimens when compared to unparasitized conspecifics. It remains to be determined if the reported effects could be a systemic result driven by parasitism or the local encysting of anisakids, as their presence was reported in the muscle of the analyzed fish (Jouini et al. 2023).

Other than hake, anisakid L3 stage may parasite a wide range of marine teleost species (Adroher et al. 1996; Mattiucci and Nascetti 2007; Debenedetti et al. 2019; Mercken et al. 2021; Ozuni et al. 2021; Martin-Carrillo et al. 2022), making them a more generalized concern for the fishing industry and the safety of consumers. Their incidence in such a diverse array of species consolidates anisakid parasites as integral components of marine ecosystems, where they play important roles in population dynamics and the shaping of community structure (Mattiucci and Nascetti 2008). Interestingly, marine parasites have been used as biological indicators in the assessment of food chain structure (Thompson et al. 2005; Lafferty et al. 2008), prevalence of pollution (Khan and Thulin 1991; Sures 2004), climate change effects (Marcogliese 2008; Palm 2011; Lõhmus and Björklund 2015), anthropogenic and environmental stresses (Landsberg et al. 1998; Jerônimo et al. 2022; Sures et al. 2023), and for fish stock assessment (Poulin and Kamiya 2015; Espínola-Novelo and Oliva 2021) and even of overall marine ecosystem health (Biswal 2020; Pérez-del-Olmo et al. 2022). Therefore, studying and understanding the intricacies of parasite-host relationships, other than supporting possible mitigation approaches, may also provide information about stress and threats to the populations of host fish. Therefore, additional studies focusing on parasite-host relationships in fish should benefit the fishing industry, in one way or the other. In fact, the importance of this topic is highlighted by its perfect framing in the United Nations’ 2030 Agenda, namely in Goals 2 (by contributing to food security and improved nutrition) and 3 (through impact on human health) (Mishra et al. 2024).

## Conclusions

This comprehensive analysis of fatty acid profiles of anisakid nematodes and adjacent muscle tissue of European hake provides a first insight into the local lipid interplay taking place between parasites and host fish, which is far from being clarified at this point. The observed disparities in fatty acid composition between parasites and their host fish in terms of fatty acid composition underscore either a competent regulation of lipid metabolism and composition or a limited acquisition of lipids from the host at this stage. Nevertheless, this characterization of the fatty acid profiles and anisakids and paratenic host tissue provides a baseline to be used for the appraisal of the adaptation of parasites to other (fish) hosts and host environments. This study is also a first step to better understand the consequences of the encysting process on the physiology and local quality of tissues in fish commonly targeted for human consumption.

The identification of characteristic fatty acid signatures in anisakids and European hake muscle tissue not only enhances our understanding of host-parasite relationships but also offers a prospective diagnostic approach for assessing parasitic infections in fish populations, as well as a prospective tool for food traceability and safety.

## Supplementary Information

Below is the link to the electronic supplementary material.

Supplementary file1 (DOCX 16 KB)

## Data Availability

No datasets were generated or analysed during the current study.
