# Supplementary material for: Evaluating fatty acid profiles in anisakid nematode parasites and adjacent tissue of European hake (Merluccius merluccius): a first insight into local host-parasite lipid dynamics
Source: Parasitol Res. 2025 Mar 13;124(3):32. doi: 10.1007/s00436-025-08477-1 (PMC11906546; doi:10.1007/s00436-025-08477-1)
Supplement: Supplementary file 1 — Supplementary file1 (DOCX 16 KB) [file 436_2025_8477_MOESM1_ESM.docx]

**Table S1:** Similarity percentage analysis (SIMPER) identifying the fatty acids (FAs) that contribute to the differences recorded in the fatty acid profiles of anisakids and adjacent hake belly flap tissue.

| **Fatty acids** | **Ind (%)** | **Cum (%)** |
| --- | --- | --- |
| 18:2*n*-6 | 14.28 | 14.28 |
| 22:6*n*-3 | 12.40 | 26.68 |
| 22:0 | 10.26 | 36.93 |
| 16:0 | 8.80 | 45.73 |
| 18:1*n*-7 | 5.86 | 51.59 |
| 18:0 | 5.32 | 56.91 |
| 22:1*n*-9 | 5.04 | 61.95 |
| 16:1*n*-9 | 3.82 | 65.77 |
| 20:2*n*-9 | 3.44 | 69.22 |
| 18:1*n*-9 | 3.07 | 72.29 |
| 20:0 | 2.37 | 74.66 |
| 10-methyl-hexadecanoate | 1.92 | 76.59 |
| 16:1*n*-7 | 1.77 | 78.36 |
| 20:1*n*-11 | 1.72 | 80.07 |
| 20:5*n*-3 | 1.69 | 81.76 |
| 20:4*n*-3 | 1.66 | 83.42 |
| 18:4b | 1.65 | 85.06 |
| 18:3*n*-3 | 1.61 | 86.67 |
| 20:1*n*-9 | 1.50 | 88.18 |
| 17:2 | 1.48 | 89.66 |
| 22:1*n*-11 | 1.48 | 91.14 |
